# Psychedelics as a treatment for disorders of consciousness

**DOI:** 10.1093/nc/niz003

**Published:** 2019-04-21

**Authors:** Gregory Scott, Robin L Carhart-Harris

**Affiliations:** 1 Department of Medicine, The Computational, Cognitive and Clinical Neuroimaging Laboratory, Division of Brain Sciences, Imperial College London, 3rd Floor, Burlington Danes Building, Hammersmith Hospital, Du Cane Road, London W12 0NN, UK; 2 Department of Medicine, Centre for Psychedelic Research, Division of Brain Sciences, Imperial College London, 5th Floor, Burlington Danes Building, Hammersmith Hospital, Du Cane Road, London W12 0NN, UK

**Keywords:** disorders of consciousness, psychedelics, psilocybin, complexity

## Abstract

Based on its ability to increase brain complexity, a seemingly reliable index of conscious level, we propose testing the capacity of the classic psychedelic, psilocybin, to increase conscious awareness in patients with disorders of consciousness. We also confront the considerable ethical and practical challenges this proposal must address, if this hypothesis is to be directly assessed.

## Introduction

Disorders of consciousness (DoC) are the most devastating form of impairment that may follow acquired brain injury. In contrast to comatose patients, those in the vegetative state (VS) and minimally conscious state (MCS) exhibit signs of wakefulness (eye opening). VS patients show no overt signs of awareness, whereas MCS patients show minimal but clearly discernible behavioural evidence of awareness. A range of therapies have been proposed for patients with DoC, including pharmacological (e.g. zolpidem, amantadine) (Gosseries *et al.* 2014), invasive- [e.g. deep brain stimulation (DBS) (Vanhoecke and Hariz 2017), vagal nerve stimulation (VNS) (Corazzol *et al.* 2017)] and non-invasive electrical stimulation [e.g. transcranial direct current stimulation (Thibaut *et al.* 2014)], and transcranial magnetic stimulation (TMS) (Pistoia *et al.* 2013). However, no treatments have consistently shown beneficial effects on conscious awareness or functional recovery (Royal College of Physicians 2013; Giacino *et al.* 2014; Vanhoecke and Hariz 2017).

**Figure 1.**
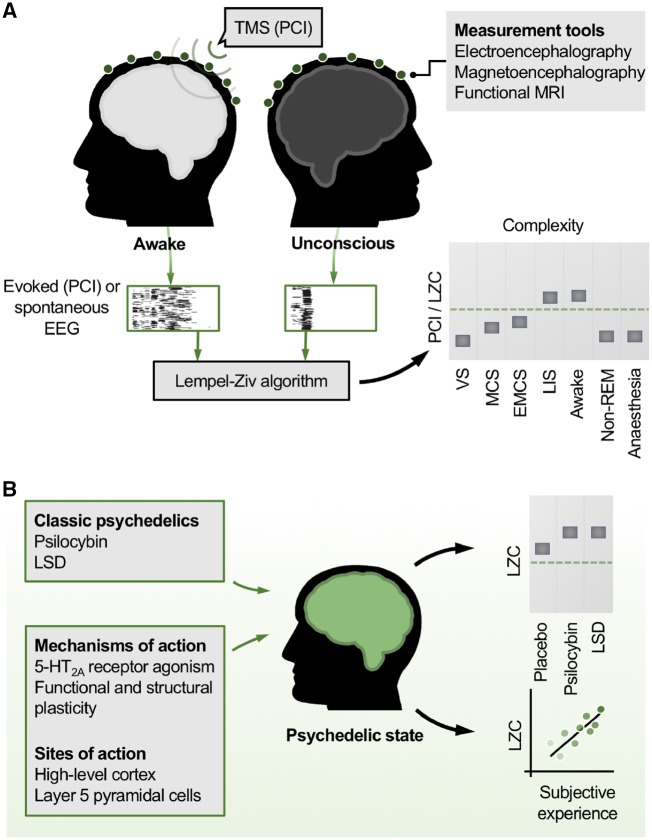
Brain complexity, consciousness and psychedelics. (**A**) Measures of brain complexity index conscious level. Empirical measures of brain complexity are high in the normal awake state and low whenever consciousness is lost. In the case of the perturbational complexity index (PCI Casali *et al.* 2013), a pulse of transcranial magnetic stimulation (TMS) provides a cortical perturbation and the evoked electroencephalogram (EEG) responses are recorded. Alternatively, spontaneous EEG data alone are recorded. The Lempel-Ziv algorithm, a measure of compressibility, quantifies the complexity (LZC) in the thresholded EEG data (illustrated by black and white grids). LZC values robustly index conscious level. VS = vegetative state; MCS = minimally conscious state; EMCS = emergence from MCS; LIS = locked-in syndrome; LZC = Lempel-Ziv complexity; non-REM = non-rapid eye movement sleep; PCI = perturbational-complexity index. (**B**) Psychedelics increase brain complexity above normal levels. Classic psychedelics increase brain complexity measures like LZC above the levels in the normal awake state. This raises the possibility that psychedelics could increase conscious awareness in patients with disorders of consciousness, where brain complexity is low. LSD = lysergic acid diethylamide.

Classic psychedelics are currently undergoing significant investigation for the treatment of a range of psychiatric disorders (Carhart-Harris and Goodwin 2017). Here, we propose that the classic psychedelic, psilocybin, be explored as a treatment to increase conscious awareness in patients with DoC. A scientific rationale is proposed based on findings from research into the neurobiology of DoC and the effects of psychedelics. Developments in these hitherto separate fields of inquiry now suggest a potential therapeutic avenue, based on the twin discoveries that measures of *brain complexity* reliably index conscious level, and that brain complexity can be increased by psychedelics (Fig. 1).

## Brain Complexity and Consciousness

Complexity is a multifaceted concept that pervades many branches of the physical and life sciences. In the neurosciences, many theoretical accounts of consciousness have related the complexity of dynamics in a neural system to the manifestation of conscious experiences (Tononi *et al.* 1994; Edelman 2009; Ruffini 2017; Carhart-Harris 2018). One influential formulation has been that of *neural complexity*, proposed by Tononi and Edelman in 1994 (Tononi *et al.* 1994). This concept accounts for two fundamental features of consciousness, namely *differentiation*, the property that any particular experience is composed of many different components and is distinguishable from any other experience, and also *integration*, the property that any given conscious experience involves the integration of components into a unified whole. Importantly, neural complexity could, in principle, be calculated empirically, as the average mutual information—a measure of information sharing—between each subset and the rest of a system. Tononi and Edelman posited that during conscious awareness, ‘heterogeneous patterns of short-term correlations within the corticothalamic system will result in [high neural complexity]’ (Tononi *et al.* 1994).

Several theories of consciousness have since been advanced that emphasize a link between different formulations of complexity within brain activity and conscious level. Alongside these theoretical developments has been the introduction of a wide range of measures of dynamical complexity. These various measures reflect the diversity of definitions of complexity in use [for review, see Arsiwalla and Verschure (2018), Seth *et al.* (2006); see also Bassett and Gazzaniga (2011), Cocchi *et al.* (2017) for broader reviews in complex systems theory] and differ in the extent to which they directly capture the properties of differentiation versus integration, as well as temporal versus spatial complexity, and in their computational feasibility for large datasets.

Despite heterogenous definitions of complexity, a prediction shared by many theories of consciousness is that complexity should be high in the normal awake state and low whenever consciousness is lost, be it through anaesthesia, non-rapid eye movement (REM) sleep, or acquired brain injury. In the past two decades, a raft of empirical support for these predictions has emerged. Massimini and colleagues have provided striking evidence in favour of the principle via use of the so-called perturbational-complexity index (PCI). PCI quantifies the complexity of electroencephalogram (EEG) responses to pulses of TMS (Fig. 1A) (Casali *et al.* 2013). This perturbational approach has been likened to hitting a bell and measuring the complexity of the reverberations that follow. The PCI has been shown to robustly index conscious level across a range of states, including wakefulness (where the PCI is highest), sedation, non-REM sleep and anaesthesia. In patients with DoC, the PCI is lowest in VS patients, followed by patients in the MCS, then those emerged from MCS (denoted EMCS). In contrast, patients with locked-in syndrome, who have intact conscious awareness but cannot respond motorically, show PCI levels as high as healthy awake subjects (Casali *et al.* 2013).

At the heart of the PCI approach is quantification of the complexity of TMS-evoked EEG responses using an implementation of the Lempel-Ziv algorithm, a measure of *compressibility* which counts the number of unique patterns in a sequence, hence its everyday use in compressing large computer files (‘zipping’). Importantly, the Lempel-Ziv complexity (LZC) measure can also be applied to EEG recordings of spontaneous brain activity, i.e. without TMS perturbation. Whilst there are substantial differences between the spontaneous and perturbational approach, particularly that PCI evaluates only the complexity of deterministic responses of the cortex to TMS (Casali *et al.* 2013), the LZC of spontaneous EEG also effectively differentiates between conscious and unconscious states [including anaesthesia (Bai *et al.* 2015; Schartner *et al.* 2015) and sleep (Schartner *et al.* 2017b)]. In DoC, LZC-based values of spontaneous EEG reliably discriminate VS from MCS patients (Wu *et al.* 2011; Sitt *et al.* 2014) and values increase monotonically with patients’ conscious level (Sitt *et al.* 2014).

Our interpretation of these spontaneous EEG results is that LZC principally captures the variability or diversity of brain activity (i.e. differentiation rather than integration), and so behaves similarly to other measures of information entropy (i.e. capturing signal diversity over time). These related entropy-based metrics also appear to track conscious level [see Schartner *et al.* (2015), Carhart-Harris (2018) for further discussion]. Please see Schartner *et al.* (2015) and Mediano *et al.* (2019) for further discussion of these topics, and note that, for the sake of disambiguation, from here on, when we refer to ‘complexity’ we are referring to the ‘differentiation’ component in the original conception of ‘brain complexity’, i.e. the component that is measurable via LZC or a related entropy-based metric.

## Psychedelics Increase Brain Complexity

Until recently, it was generally assumed that, in terms of states of consciousness, brain complexity would be maximal during normal wakefulness, since all other tested states of reduced consciousness (e.g. non-REM sleep, anaesthesia, DoC) feature correspondingly lower complexity values. It was therefore remarkable to discover that brain complexity values recorded during the psychedelic state exceed those found in normal waking consciousness (Fig. 1B). Specifically, in human subjects, increases in brain complexity (LZC) in excess of those seen in normal wakefulness were observed with psilocybin, lysergic acid diethylamide (LSD) and ketamine (at ‘psychedelic-like’ doses) (Schartner *et al.* 2017a). This finding has been replicated using a variety of complexity measures and measurement tools, including EEG, magnetoencephalography and functional MRI [see Carhart-Harris (2018) for review]. Furthermore, the magnitude of complexity increases correlated with the subjective intensity of the psychedelic experience (Schartner *et al.* 2017).

## Increase Complexity, Increase Conscious Awareness?

Given that impairments in conscious awareness appear to closely relate to reductions in measures of brain complexity and psychedelics robustly increase brain complexity, could psychedelics elevate conscious awareness in patients with DoC*?*

Note that this hypothesis does not require that brain complexity be the *cause* of conscious awareness. Brain complexity *per se* may rather, in the terminology of Seth and Edelman (2009), be an *explanatory correlate* of the neural processes intimately related to conscious awareness. With this qualification in mind, a key question for our proposal is whether it is possible to increase measures of brain complexity without increasing conscious awareness. If it were possible, then this would negate our hypothesis and call into doubt the relationship between consciousness and brain complexity, at least as we define it here.

The classic psychedelic, psilocybin, is currently undergoing substantial clinical investment (Carhart-Harris and Goodwin 2017). Psilocybin is a prodrug of psilocin (4-hydroxy-dimethyltryptamine), whose principal psychoactive effects appear to be mediated by serotonin 2A (5-HT_2A_) receptor agonism. Psilocybin elevates measures of brain complexity in healthy humans (Schartner *et al.* 2017a; Carhart-Harris 2018) and many other lines of evidence support the idea that psilocybin could elevate conscious awareness in patients with DoC. The 5-HT_2A_ receptors have their densest expression in the high-level cortical areas belonging to the default-mode network, which has been strongly implicated in conscious processing as well as the psychedelic state (Guldenmund *et al.* 2012; Beliveau *et al.* 2017; Carhart-Harris 2018). Most 5-HT_2A_ receptors are expressed post-synaptically on layer 5 pyramidal neurons (Berthoux *et al.* 2018). These large, deep layer neurons are known to be key integration units in the cortex, and are the only cell type with dendrites spanning all cortical layers (Shai *et al.* 2015). In addition, presynaptic 5-HT_2A_ receptors located at thalamo-cortical synapses have been shown to play an important role in the control of thalamo-frontal connectivity, also known to be important for consciousness (Giacino *et al.* 2014; Barre *et al.* 2016). 5-HT_2A_ receptor agonism in animals is associated with enhanced cognitive flexibility as well as cortical neural plasticity (Frankel and Cunningham 2002; Boulougouris *et al.* 2008; Furr *et al.* 2012; Zhang and Stackman 2015; Ly *et al.* 2018; Olson 2018) whereas 5-HT_2A_ receptor antagonism is associated with reduced cognitive flexibility and increased slow-wave sleep (Carhart-Harris and Nutt 2017). We recognize that one should be cautious when extrapolating from findings in animals to humans. However, there is some tentative evidence that cognitive flexibility is also enhanced in humans under psychedelics (Kuypers *et al.* 2016), although we would be hesitant to infer from this that psychedelics can enhance cognitive performance [see also Bayne and Carter (2018) and Carhart-Harris and Nutt (2017)].

## Complexity, Conscious Content and Arousal

The standard conception of consciousness is that it encompasses two inter-related dimensions (Laureys *et al.* 2009; Boly *et al.* 2013): (i) the ‘content’ of consciousness, thought to be primarily related to cortical mechanisms, and (ii) wakefulness, or arousal, which subserves (i) and is controlled by the ascending activation systems of the brainstem and basal forebrain (i.e. the reticular activating system) (Boly *et al.* 2013). A key question is: how do these dimensions relate to measures of brain complexity like LZC?

Studies of impaired consciousness suggest that LZC and related measures of complexity chiefly index conscious *content* rather than *arousal*, e.g. as shown by the reductions in LZC that differentiate VS from MCS patients (Casali *et al.* 2013; Sitt *et al.* 2014) and non-REM from REM sleep (Abásolo *et al.* 2015). To our knowledge, there is no evidence that stimulant drugs, such as D-amphetamine or methylphenidate, which primarily increase arousal, increase brain complexity measures. In the case of psychedelics, our own experience is that arousal provides minimal explanatory value for describing the quality of the psychedelic experience. Moreover, we have argued that the evidence overwhelmingly suggests that psychedelic-related elevations in LZC or information entropy (to which LZC is closely and formally related) reflect an increased richness of conscious experience (Carhart-Harris 2018). Together, these observations suggest that targeting increases in conscious content, rather than (or perhaps in addition to) arousal, may be key to increasing conscious awareness in DoC patients.

Experiments comparing psychedelics with stimulant medications may help address the question of whether drugs presupposed (here) to increase conscious content (e.g. psilocybin) have more significant effects on brain complexity, and conscious content, than drugs that primarily promote arousal. Neurotransmitter systems implicated in the regulation and maintenance of arousal include noradrenaline, dopamine, acetylcholine, orexin, adenosine, histamine and 5-HT (Boutrel and Koob 2004; Ciurleo *et al.* 2013; Mura *et al.* 2014). Most classic stimulants act on catecholamines, and drugs such as D-amphetamine (Zhang *et al.* 2009), levodopa (Krimchansky *et al.* 2004) and modafinil (Dhamapurkar *et al.* 2017) have been used in DoC patients, with evidence of modest and variable clinical effects [see Ciurleo *et al.* (2013) and Mura *et al.* (2014) for review]. Our working hypothesis is that psilocybin is able to enhance conscious awareness to a greater extent than these stimulant-based alternatives.

## The Relevance of a Multidimensional Conception of Consciousness

The current classification of DoC uses a taxonomy of states of consciousness ordered along a single scale, i.e. with EMCS patients having a higher level of consciousness than MCS patients who, in turn, have a higher level of consciousness than VS patients. However, recent challenges to this standard unidimensional construct of ‘levels of consciousness’ have been proposed (Bayne *et al.* 2016, 2017; Bayne and Carter 2018). These commentaries argue that the full range of consciousness-related capacities would be better classified using a graded, multidimensional space that captures, e.g. cognitive, sensory, affective and behavioural characteristics (Bayne *et al.* 2017). The same criticism has been levelled to applying the ‘levels of consciousness’ construct to all global states of consciousness—e.g. alert wakefulness, REM sleep, general anaesthesia, absence seizures and the psychedelic state—in that it fails to do justice to the evidently multifaceted nature of these states (Bayne *et al.* 2016; Bayne and Carter 2018). We are sympathetic to this view but also mindful of the pragmatic value of simple guiding principles in science. Thus, it remains to be seen how such a multidimensional framework, the details of which remain somewhat underspecified (Bayne *et al.* 2016), will align with the unidimensional complexity measures such as PCI and LZC that dominate empirical studies of states of consciousness and indeed current theories of consciousness (Baars 2005^;^Tononi *et al.* 2016; Carhart-Harris 2018). As we acknowledged earlier, we see our proposal (to explore psychedelics as a treatment in DoC) as a challenge to the unidimensional conception of conscious level as indexed by brain complexity, in that to find a dissociation between complexity increases and conscious awareness would suggest important limitations to this simplistic framework.

## An Ethical Hypothesis?

We believe that the evidence presented here suggests a strong scientific case for research exploring the hypothesis that psychedelics can increase conscious awareness in patients with DoC. However, stern ethical objections could supervene to prevent it from being tested. Ethical consideration of any interventional research in patients with DoC must grapple with dual opposing imperatives: on the one hand are concerns about risks of harm to patients lacking the capacity to consent; and on the other hand, is the principle that research must be done if we are ever to progress in our ability to improve the health of these patients. We agree with others who have argued that the inability of patients to consent doesn’t make research ethically *illegitimate* so long as it is ethically *proportionate*, a judgement that hinges on the accurate assessment of risks and benefits (Giacino *et al.* 2014; Fins *et al.* 2008).

Contrary to the alarmist campaigning that so negatively affected perceptions of psychedelics during and after the 1960s, plant-based psychedelics have been used for centuries for therapeutic purposes, and a recent resumption of clinical research with them has established conditions for their safe administration. Psilocybin has a particularly favourable safety profile, with a low toxicity and addiction potential (Passie *et al.* 2002; Carhart-Harris and Goodwin 2017). Evidence clearly indicates that, contrary to a popular misconception, psychedelics, when used with the relevant safeguards in place, are associated with positive rather than negative long-term mental health outcomes (Carhart-Harris and Goodwin 2017). There is now converging support for the safety and tolerability of psilocybin in a variety of psychiatric disorders [e.g. see Carhart-Harris and Goodwin (2017) for review]. The psychedelic effects of psilocybin are detectable 30–60 min after oral dosing (10–25 mg), peaking at 2–3_** **_h, and subsiding to negligible levels at least 6_** **_h post-dose (Carhart-Harris *et al.* 2016). Intravenous administration accelerates the onset into the domain of seconds and shortens the duration of the experience considerably (Turton *et al.* 2014).

Several experimental interventions in DoC patients have been invasive by comparison with what propose here. For example, the surgical implantation of DBS electrodes has been carried out for 50_** **_years, despite a lack of consistent evidence of benefits for improving conscious awareness (Vanhoecke and Hariz 2017). Recently, VNS implantation has been reported in a single case of a VS patient. Only modest behavioural improvements were observed when stimulation levels were titrated over a 6-month period (Corazzol *et al.* 2017).

A special ethical concern for neuromodulatory treatments such as DBS and VNS has been the possibility of a ‘self-awareness paradox’, whereby through an increase in conscious awareness, the patient experiences a concomitant increase in awareness of his/her clinical predicament and disability (Schiff *et al.* 2009). For psilocybin, treatment could conceivably also induce a transient unpleasant state of awareness, sometimes referred to colloquially (although not always accurately or helpfully) as a ‘bad trip’. It is difficult to gauge the likelihood or nature of either scenario in DoC patients given a psychedelic. A low baseline level of awareness might intuitively imply that unpleasant psychological phenomena will be both less likely and less severe than in fully aware subjects. Our experience is that such phenomena are rare in the investigational context, but more likely at higher doses and in settings lacking in psychological support.

Based on experience and accumulating evidence, there appear to be ways to mitigate the risks of difficult psychological experiences (Carhart-Harris *et al.* 2018). In our Phase 2 study in treatment-resistant depression, psychologically supported administration of oral psilocybin (10–25 mg) was well-tolerated by all patients, with the most common adverse events being mild transient anxiety just prior to as well as during drug onset (Carhart-Harris *et al.* 2016). In the case of DoC patients, one might expect anticipatory anxiety to be lower than in neurotypical individuals and the risk of anxiety could be further reduced by careful attention to the environment and ensuring familiar carers are at hand during dosing.

## Future Horizons

The modest outcomes from previous interventional studies in patients with DoC should temper optimism that psilocybin could bring clinically meaningful benefits, particularly in cases of extensive neuronal loss, e.g. a sufficient degree of functional neuronal architecture may need to be in place for psychedelics to elicit a functionally meaningful effect. However, we believe that pragmatics and need, supported by sound theory and evidence, as well as proper consideration of ethics and care, should dictate how to proceed.

One potential starting point might be to test the idea using animal models, but we are doubtful of its translational value to our hypothesis. While animal studies have informed our understanding of the neural circuitry involved in information processing in general (Boly *et al.* 2013), it is less evident that any existing animal model of acquired brain injury (i.e. either severe traumatic brain injury or adult hypoxic ischaemic injury) holds relevance for the goal of understanding the recovery of conscious awareness in human individuals with DoC. An alternative to injury models would be to test in sedated animals whether psilocybin increases measures of brain complexity from a baseline of sedative-induced reduced complexity. Intriguingly, evidence of this kind can be found in the literature from the 1950s, with a report that LSD reverses the sedating effects of anaesthetic doses of barbiturates in cats (Apter 1958).

Given the difficulties in assessing consciousness in non-human animals, an advance on this would be to carry out the experiment in sedated healthy human volunteers, measuring complexity with scalp EEG and either LZC on spontaneous EEG signals or using PCI, accompanied by repeated behavioural measurements of consciousness, before and after psilocybin administration. Moreover, by combining spontaneous EEG/LZC and PCI measures within the same sample, one could potentially gain insight into their inter-relatedness or indeed separation and differential relevance for conscious awareness.

Whilst there are fundamental difficulties in extrapolating findings from sedated volunteers to patients with DoC, positive findings would support the case for a study of psilocybin in DoC patients. A related experiment in humans could be carried out in sleep, testing the hypothesis that psychedelics increase complexity and conscious level in non-REM sleep, perhaps by promoting REM sleep, evidence for which can also be found in the historical literature (Muzio *et al.* 1966; Torda 1968). Experiments comparing psilocybin with stimulant medications would help answer the question of whether drugs presupposed to increase conscious content (e.g. psilocybin) have more significant effects on brain complexity and conscious awareness than drugs that more specifically promote arousal.

## Experimental Considerations

Assuming the scientific, ethical and regulatory case can be won for the testing of our hypothesis, we suggest some principles for the design of preliminary studies of psilocybin in patients with DoC, based on our experience of psychedelic experimental research.

We would advocate an incremental and adaptive approach, where the first steps would be to establish safety and tolerability and examine the signal changes of interest (i.e. changes in LZC in spontaneous EEG activity). This step-wise procedure would then be followed by a focus on optimizing the dosage parameters, and measuring and searching for the desired behavioural effects, i.e. an observable increase in conscious awareness, while maintaining good tolerability.

We expect that the patient inclusion criteria and recruitment protocol would be similar to previous early-phase pharmacology studies in this population. Exclusion criteria should include a history of psychotic disorder, as is typical with psychedelic research, and an abnormal resting electrocardiogram. Serotonergic antidepressants have been found to downregulate the 5-HT_2A_ receptor, and attenuated responses to psychedelics have previously been reported in individuals chronically medicated with serotonergic antidepressants (Bonson *et al.* 1996). We would therefore exclude any patients receiving these drugs or request controlled washout from these medications, for which we have a working protocol.

As outlined above, we would first aim to test the hypothesis that psilocybin increases measures of brain complexity. To do this safely, we would use a dose-escalation design, using low doses to assess tolerability before moving to a higher, potentially therapeutic dose range. Doses in the range of 25–40 mg are used clinically and have been found to induce profound, existentially ‘transformative’ experiences in both healthy and clinical populations. The physiological safety of 30_** **_mg/70_** **_kg psilocybin has been well-demonstrated in healthy volunteers (Griffiths *et al.* 2006). We would therefore aim to reach such doses, particularly as it is possible DoC patients have reduced sensitivity to the effects of psilocybin. Although it would add complication to procedures, 5-HT_2A_ receptor positron emission tomography could allow this assumption to be tested empirically (Kumar and Mann 2014).

We would, as a minimum, record continuous scalp EEG before, during and after dosing, and calculate LZC measures of brain complexity offline. If practicable, it would be beneficial to additionally use the PCI (i.e. EEG combined with TMS) (Casali *et al.* 2013). Alongside neurophysiological measures, we would also record repeated cardiorespiratory observations (heart rate, blood pressure, respiratory rate) and carefully observe the participant for signs of psychological distress and increased sympathetic nervous system activity. For an early-phase study, although behavioural endpoints would not be of primary interest, standardized assessments would be incorporated where this is feasible (i.e. the Coma Recovery Scale-Revised and/or Wessex Head Injury Matrix).

In subsequent analysis, it would be of interest to explore the relationship between pre- and post-dose EEG complexity and behavioural measures. Given the evidence that measures derived from EEG connectivity in patients with DoC could not only prognosticate recovery (Sitt *et al.* 2014; Chennu *et al.* 2017), but also predict response to intervention (Thibaut *et al.* 2018), it is possible that pre-dosage EEG measures could be used in a similar way in relation to psychedelic treatment.

We recommend detailed consideration of the choice and setup of the environment and the support provided to the patient during study sessions, although we acknowledge it will be difficult to know how a DoC patient could be prepared for the expected effects of the drug. The sessions themselves would be carried out in a familiar environment for the patient, with their normal carers present. In our study of treatment-resistant depression, psychological support was provided before, during and after each session. There is evidence to suggest that control of context is important for positive therapeutic responses (Carhart-Harris *et al.* 2018). The timing of dosing in relation to markers of the circadian rhythm, which is likely to influence arousal, should also be factored in (Blume *et al.* 2017).

In later studies of psilocybin as a treatment for patients with DoC, behavioural indices like the Coma Recovery Scale - Revised (CRS-R) must ultimately be used as the primary outcome measure. In this sense, we would wish to treat psilocybin no differently than any other experimental intervention for DoC. At present, we view brain complexity as an informative index of conscious content, but for this to be functionally relevant in the context of psychedelic interventions in DoC, it would be vital to establish that a psychedelic-induced increase in complexity ultimately translates into behavioural improvements, with the important caveat that it may be necessary in fact to test for covert awareness, i.e. identifying consciousness in the absence of any behavioural response, e.g. by using covert command-following paradigms (Owen *et al.* 2006).

The growing evidence for pro-plasticity effects via 5-HT_2A_ receptor agonism suggests that later placebo-controlled studies could be designed to test whether a given dose regimen may enhance standard rehabilitative care, with the aim of augmenting its effectiveness. Here, a ‘micro-dosing’ strategy could be considered, similar to that adopted by many current studies, whereby threshold perceptible doses of psychedelics are given two to three times per week, and acute psychological ‘side effects’, such as anxiety, may be minimal. Thus, a micro-dosing protocol would put more focus on drug-assisted rehabilitative care rather than neuropharmacology alone, which would be consistent with the therapeutic model currently being employed in psychiatric contexts.

Overall, we propose an explorative, adaptive approach (within specified boundaries, and with an emphasis on caution) for this research, as so much is uncertain, including of course, positive eventualities.

## Conclusion

Disorders of consciousness present unique prospects for fundamental scientific discovery and major clinical breakthroughs but also significant ethical and pragmatic challenges. The nascent renaissance in psychedelic research has shined a light on the study of consciousness, revealing anomalous positive effects on the complexity of brain activity, the low values of which have come to define states of impaired consciousness. The modern era of responsible scientific experimentation with psychedelics is yielding significant support for their safety across a range of conditions. Taken together, we call for an open-minded attitude about the possibility of exploring the potential of psychedelics to elevate conscious awareness in patients with DoC.

## Authors’ Contributions

G.S.: conceptualization, drafting of and revision of the manuscript; R.L.C.-H.: conceptualization and revision of the manuscript.
